# Rho GTPase Cdc42 Is a Direct Interacting Partner of Adenomatous Polyposis Coli Protein and Can Alter Its Cellular Localization

**DOI:** 10.1371/journal.pone.0016603

**Published:** 2011-02-02

**Authors:** Thankiah Sudhaharan, Wah Ing Goh, Kai Ping Sem, Kim Buay Lim, Wenyu Bu, Sohail Ahmed

**Affiliations:** Neural Stem Cell Laboratory, Institute of Medical Biology, Singapore, Singapore; University of Birmingham, United Kingdom

## Abstract

Adenomatous Polyposis Coli (APC) is a tumor suppressor gene product involved in colon cancer. APC is a large multidomain molecule of 2843 amino acid residues and connects cell-cell adhesion, the F-actin/microtubule cytoskeleton and the nucleus. Here we show that Cdc42 interacts directly with the first three armadillo repeats of APC by yeast two-hybrid screens. We confirm the Cdc42-APC interaction using pulldown assays *in vitro* and FRET assays *in vivo*. Interestingly, Cdc42 interacts with APC at leading edge sites where F-actin is enriched. In contrast, Cdc42 interacts with the truncated mutant APC^1–1638^ in cellular puncta associated with the golgi-lysozome pathway in transfected CHO cells. In HCT116 and SW480 cells, Cdc42 induces the relocalization of endogenous APC and the mutant APC^1–1338^ to the plasma membrane and cellular puncta, respectively. Taken together, these data indicate that the Cdc42-APC interaction induces localization of both APC and mutant APC and may thus play a direct role in the functions of these proteins.

## Introduction

Adenomatous Polyposis Coli (APC) is a tumor suppressor gene product involved in cellular processes including Wnt signaling, cell growth, cell fate determination and cell migration. APC is a multidomain scaffold protein of 2843 amino acid (aa) residues linking the nucleus to cell surface events through associations with the cytoskeleton [1]. The APC N-terminal is a region that contains seven armadillo repeats [2] through which it binds KAP3 (kinesin-associated protein 3), Asef (Cdc42/Rac-specific guanine nucleotide exchange factor) and IQGAP1 (IQ motif containing GTPase activating protein 1) [3]–[5]. The central domain has a mutation cluster region that binds axin and beta-catenin/alpha-catenin [2], [6], [7]. The C-terminal region of APC consists of positively charged amino acid residues responsible for binding microtubules and EB1 (end-binding protein 1). The binding site for a PDZ domain [8] is the last domain of the C-terminus.

Mutation of APC causes Familial Adenomatous Polyposis, an inherited form of colon cancer. The most frequently found truncations are divided into two groups. Type I truncations, which are the most common type among sporadic colorectal tumors, retain the N-terminal nuclear export signal (n-NES) but lack the C-terminal NES (c-NES). Type II APC truncations retain the n-NES as well as the 5′-most c-NES. Interestingly, type II APC truncations show the APC protein to be excluded from nuclei [9]. Truncations lacking the C-terminal domains of APC are the ones associated with tumorigenesis. Germline mutations occurring at codons 1061 and 1309 account for a third of the mutations. Apart from these hotspots, germline mutations in APC are spread fairly uniformly between codons 200 and 1600 but rarely occur beyond codon 1600. Truncated APC proteins found in colorectal tumors seldom retain the basic domain [10], [11].

Tumor formation and metastasis involve coordinated changes in the actin and microtubule cytoskeletons [12], [13]. Overexpression of Rho GTPases is likely to increase tumor invasiveness through effects of cell migration and matrix degradation [14], [15]. The small GTP-binding protein Cdc42 regulates cell morphology and polarity signals by interacting directly with a number of protein effectors. For example, Cdc42 regulates the localization and function of IRSp53 (insulin receptor substrate protein 53 kD) leading to complex formation with N-WASP (neural Wiskott-Aldrich syndrome protein), Mena (mammalian enabled), and Eps8 (EGF receptor kinase substrate 8) to induce filopodium formation [16]–[18]. Investigation of protein complexes that link actin, microtubules and APC is likely to reveal important elements of tumor formation. The Cdc42 effector IQGAP1 interacts directly with APC and may be responsible for actin accumulation at the leading edge and directional migration [5]. Cdc42 controls cell polarity through the spatial regulation of GSK-3β (glycogen synthase kinase-3β) phosphorylation of APC [19]. APC has been found to interact with Asef with a potential consequence for cell migration [20].

In the present study, we carried out a yeast two-hybrid (Y2H) screen of nine cDNA libraries using Cdc42 as a bait, which yielded 10 novel interactors including a 431 aa fragment of APC that contains the first three armadillo repeats. The Cdc42 interaction with this 431 aa fragment was further confirmed using affinity pulldowns, FRET [18] and fluorescence lifetime imaging microscopy (FLIM; [21]). Cdc42 was also found to interact directly with both full-length APC and a truncation mutant APC^1–1638^, which is implicated in colon cancer. In CHO cells Cdc42 interacts with APC at the leading edge and with APC^1–1638^ in cellular puncta. Interestingly, Cdc42 induced the relocalization of APC^1–1638^ from cellular puncta to the late endocytic pathway. In contrast, Cdc42 induces relocalization of full-length APC to the leading edge with F-actin. Furthermore, Cdc42 induced the relocalization of endogenous APC and APC^1–1338^ (the most commonly used mutant for endogenous studies) to the leading edge and cell puncta in HCT116 and SW480 cells, respectively. Taken together, these data suggest that Cdc42 may play an important physiological role in the function of APC and APC mutants by binding these proteins directly and influencing their cellular localization.

## Materials and Methods

### Plasmids

mRFP is a kind gift from Prof. Roger Y. Tsien (University of California, San Diego). GFP-Cdc42V12, HA-Cdc42 and GFP-Cdc42N17 were generated by subcloning the respective inserts into the pXJ40 vector. mRFP-Cdc42V12 and mRFP-Cdc42N17 were created by subcloning the respective gene into the pXJ40-mRFP vector in between the BamHI and BglII restriction sites. mRFP-APC^222–653^ was made by inserting the amino acid sequences of APC^222–653^ into the pXJ40-mRFP vector in between the BamHI and NotI restriction sites. APC^222–452^ and APC^453–653^ were made by inserting the corresponding amino acid sequences of APC into the pXJ40-mRFP vector in between the BamHI and NotI restriction sites (generously given by Yong Hwee Foo). mRFP-GFP tandem fusion was made by inserting GFP between the BamHI and NotI sites in the pXJ40-mRFP vector. mRFP-SBP-Cdc42V12 and mRFP-SBP-GFP were made by inserting streptavidin binding protein (SBP) into the BamHI site. Full-length GFP-APC and GFP-APC^1–1638^ were a kind gift from Dr. Maree Faux (Ludwig Institute for Cancer Research, Australia). All restriction enzymes were obtained from NEB Biolabs.

### Yeast two-hybrid screening

Matchmaker™ pre-transformed human cDNA libraries were screened using the Matchmaker™ GAL4 two-hybrid system 3 according to the manufacturer's protocol (Clontech). Briefly, the bait protein Cdc42Q61L/C189S cDNA was cloned into the pAS2-1 vector and the resulting construct transformed into AH109 strain yeast cells. Transformants were selected for growth on synthetic dropout (SD) medium lacking tryptophan, and mated with Y187 strain yeast cells pretransformed with human cDNA libraries. Putative positive bait-prey interactions in the resulting diploids formed were identified by growth on quadruple dropout (QDO; SD medium lacking adenine, histidine, leucine and tryptophan) medium. Yeast plasmid DNA was extracted from clones that grew on QDO medium, electroporated into *Escherichia coli* KC8 strain cells, and the bacterial plasmids isolated and sequenced. Sequences obtained were analyzed by BLAST to determine the identity of the putative Cdc42-binding proteins. Complementation assays to confirm the putative interactions in a clean yeast background were done by transforming bait and prey plasmids into AH109 and Y187 yeast strains respectively, and mating the transformants obtained (identified by their ability to grow on tryptophan- or leucine-deficient SD medium). The β-galactosidase assay was used to verify positive bait-prey interaction in diploids that survived on QDO medium, as well as lack of interaction in diploids that grew on SD medium lacking tryptophan and leucine but not on QDO. All yeast and bacterial strains, vectors and cDNA libraries were from Clontech, and all yeast culture media components were from Clontech and Difco and prepared according to the Matchmaker™ GAL4 two-hybrid system yeast protocols handbook (Clontech).

### Tissue Culture

CHO cells were obtained from ATCC (Manassas,VA) and grown in 75 cm^2^ tissue culture flasks up to 90% confluency in complete growth medium, in 1× F-12 Nutrient mixture (Kaighn's modification) media containing 10% fetal bovine serum (FBS) and 1% antibiotics (penicillin and streptomycin). All tissue culture reagents were obtained from Invitrogen (Singapore). CHO cell transfections were carried out by seeding the cells in a six-well tissue culture plate containing 18×18 mm pre-washed sterilized glass coverslips at 1.5×10^5^ cells/well one day before transfection. The seeded cells were transfected using Fugene6 reagent (Roche Applied Bioscience) with 0.5–1 µg of plasmid DNA. HCT116 and SW480 colon cancer cells were a kind gift from Dr. Haihe Wang (Institute of Molecular and Cell Biology, Singapore). HCT116 and SW480 cells were maintained in RPMI medium and Lipofectamine2000 (Invitrogen) transfection reagent was used to transfect both colon cancer cell lines.

### Co-immunoprecipitation assay for endogenous APC in SW480 cells

cDNA encoding mRFP-SBP-GFP (negative control) and mRFP-SBP-Cdc42V12 (experiment) were separately transfected into SW480 cells and left to express for 24 h. Cells were then lysed using 20 mM HEPES pH 7.3, 1% Triton X-100, 100 mM NaCl, 20 mM MgCl_2_, 1 mM Dithiothreitol, 1 mM sodium orthovanadate, 1 mM phenylmethylsulfonyl fluoride and complete protease inhibitor mix (Roche). The SBP tag present within the fusion protein enabled their pulldown using streptavidin-sepharose beads, followed by washing with lysis buffer, elution by boiling in SDS sample buffer and resolution on a 6% SDS-PAGE gel. Protein transfer onto PVDF membranes was mediated by a semi-dry transfer apparatus using transfer buffer of 48 mM Tris base, 39 mM glycine, 0.37% SDS and 5% methanol overnight at 4°C, with a constant current of 50 mA and voltage limit of 15 V. The blots were analyzed for the presence of APC with Ab-5 antibody (Calbiochem, 1∶100) and subsequently stripped and reprobed with streptavidin-HRP antibody (1∶1000) to assess the amounts of mRFP-SBP-GFP and mRFP-SBP-Cdc42V12.

### AP-FRET measurement

Acceptor photobleaching-FRET (AP-FRET) was measured as described in [18] by making necessary settings in a Zeiss LSM 510 confocal microscope with a C-Apochromat 63×1.2 W objective. Briefly, the transfected GFP/mRFP fusion proteins (as described earlier) were washed 24 h after transfection with 1× PBS three times and fixed with 4% paraformaldehyde (Sigma-Aldrich) for 15 min. The fixed coverslips were mounted on glass slides using Hydromount (National Diagnostics). The mounted cells were excited using 488 and 561 nm laser lines as excitation sources, by selecting the 405/488/561 dichroic mirror and the 490/565 secondary dichroic mirrors for GFP and mRFP emission respectively. The emission was monitored by selecting GFP (BP 505–550) and Red (LP 575) emission filters to record the fluorescence intensity. Region of interest (ROI) was selected and photobleached using 70% of 561 nm laser power by selecting 50 iterations. The increase in GFP fluorescence intensity followed by mRFP bleaching and measured as FRET. Percentage FRET efficiency (%FE) was calculated using the change in background subtracted fluorescence intensity as 100×[(post-bleach intensity)-(pre-bleach intensity) / (post-bleach intensity)]. Average AP-FRET value was obtained from 7–10 cells and all experiments were repeated three times. In order to verify that the increase in GFP intensity was not due to an artifact we obtained the Pearson product moment correlation coefficient (CC) r, a dimensionless index that ranges from −1.0 to 1.0 inclusive, which reflects the extent of a linear relationship between the two fluorescence intensity data of GFP and mRFP on acceptor bleaching.

### FLIM-FRET measurement

The frequency domain FLIM experiments were performed with the LIFA system (Lambert Instruments, The Netherlands) on an inverted widefield fluorescence microscope (Olympus IX71, Center Valley, PA) with a 60×1.35 oil immersion objective [21]. The excitation source was a 4 mW 470 nm LED. The emission was collected with a 490–550 nm GFP filter. FITC was used as a lifetime reference with a set lifetime of 4.0 ns. Twelve phase and modulation shifted images were taken and fitted with a sinus function using software supplied by the manufacturer. The phase shifted intensity data was used for lifetime calculation. Transiently transfected and fixed CHO cells (as described above) were used to obtain the FLIM image. GFP-fusions of the respective interacting partners (donor alone) were used to obtain the control lifetime. The percentage FLIM-FRET efficiency was calculated as FRET = 100×[1−(lifetime of donor with FRET/lifetime of donor without FRET)]. Several ROI were drawn on the images (7–10 cells) and the average lifetime and standard deviation calculated. All experiments were repeated three times.

### Colocalization analysis

For colocalization analysis we used either the Zeiss 510 or Olympus FV1000 microscope. Fluorescence images of fixed cells were obtained in either two channel or three channel settings. All images were obtained sequentially with a constant offset and pinhole setting in all channels. Colocalization analysis was carried out using Zeiss or Olympus software. CHO cells were transfected with GFP-APC alone or together with HA-Cdc42V12 or mRFP-Cdc42V12 and fixed as described earlier. Cells coexpressing GFP-APC with or without HA-Cdc4242 or HA-Cdc42N17 were permeabilized with 0.1% Triton X-100 in PBS for 5 min. Cells were blocked with 3% BSA for 30 min at room temperature and incubated with anti-HA mouse monoclonal primary antibody (1∶100) in blocking solution at room temperature for 60 min. Cells were further washed three times with PBS and then incubated with Alexa 405 antibody (1∶200) along with diluted (1∶50) TRITC-labeled phalloidin (Molecular Probes) at room temperature for 60 min and washed three times with PBS. Cells expressing mRFP-Cdc42V12 were treated with FITC-labeled phalloidin (Molecular Probes) at room temperature for 5 min. The cells were then mounted as described earlier and visualized using confocal fluorescence microscopy.

### Immunofluorescence analysis

#### (a) For golgi, lysozomes and microtubules

CHO cells transiently transfected with respective plasmids were fixed and immunostained as described earlier, using the following primary antibodies; anti-Lamp1 (goat polyclonal, 1∶100, Santa Cruz) for lysozome, anti-HA (mouse monoclonal, Millipore) for HA-Cdc42V12 or HA-Cdc42N17, GM130 (mouse monoclonal, BD) for golgi and anti α-tubulin (mouse monoclonal, 1∶500, Sigma) for microtubules, followed by incubation with the respective secondary antibodies (Molecular Probes) at 1∶200 dilution, anti-mouse Alexa 405 and anti-goat Cy5 for lysozome-Cdc42V12 colocalization, anti-mouse Alexa 405 for golgi and anti-mouse Alexa 568 for microtubules.

#### (b) Endogenous APC detection

HCT116 and SW480 cells, either untransfected or transfected with mRFP-Cdc42V12 or Cdc42N17 (alone or coexpressed with GFP-actin), were fixed and blocked as described previously. Endogenous APC was stained as described earlier using primary antibodies targeting mouse APC (ab-2 for HCT116 cells and ab-5 for SW480 cells; 1∶50 Calbiochem) followed by incubation with anti-mouse Alexa 488 secondary antibody (1∶200, Molecular Probes). For GSK-3β inhibitor studies, HCT116 cells transfected with mRFP-Cdc42V12 were treated with 10 µM cell-permeable PKCζ–specific pseudosubstrate (Calbiochem) for 1 h at 37°C, followed by APC immunostaining.

## Results

### Cdc42 interacts with APC^222–653^

Y2H screening is an *in vitro* genetic approach to identifying binding partners from a library of polypeptides. We used the GAL4 Y2H system to identify protein partners of Cdc42. Human cDNA libraries were screened using a double mutant form of Cdc42 (Cdc42Q61L/C189S) as bait. The Q61L and V12 both mutations of Cdc42 render the protein GTPase-deficient, thus increasing affinity for effector proteins, which interact only with the active GTP-bound form of Cdc42. This also enables the exclusion of GEFs and GDIs, which interact with the inactive GDP-bound form of Cdc42. The C189S mutation overcomes the lethal effects of the Q61L mutation by disrupting the isoprenylation site in Cdc42, preventing membrane localization of the protein and making it biologically inactive. This mutation also favours the localization of the bait protein to the nucleus, which is essential for the Y2H system to work.

Nine human cDNA libraries (brain, fetal brain, bone marrow, heart, liver, kidney, ovary, testis and 11-day embryo) were screened, and putative bait-prey interactions gave rise to yeast clones that survived on QDO medium. pACT2 plasmids were isolated from 325 of these clones and their sequences determined and matched against entries in the NCBI NR (non-redundant) nucleotide sequence database using BLAST in order to identify the potential Cdc42 target proteins that they encoded. Of the 325 sequences, 247 matched to existing database entries (known proteins), while 46 were novel sequences not present in the database. Of the remainder, four matched to uncharacterized cDNA, two to RPCI-11 BAC library clones, 11 to chromosomal regions, five to repetitive sequences and 10 were found to be false positives. Interestingly the brain library yielded the largest proportion of clones (Fig. S1 (i)) with many known Cdc42 interactors represented. Among the positive interactors were known Cdc42 binding partners that contain CRIB (Cdc42/Rac-interactive binding) motifs, such as N-WASP (14 clones from brain library), ACK1 (Cdc42-activated tyrosine kinase 1; six clones from brain library) and PAK2 (p21-activated kinase 2; five clones from 11-day embryo, heart and testis libraries), as well as known Cdc42 interactors that contain CRIB-like motifs, such as IRSp58 (50 clones from brain library), Par6 (par-6 partitioning defective 6 homolog alpha; 15 clones from brain and testis library), IRSp53 (12 clones from brain, fetal brain, kidney, liver and testis libraries; [22]) and Cdc23 (cell division cycle protein 23; four clones from brain and testis libraries; [23]).

Sixty-one clones encoded a total of 26 proteins not previously known to bind to Cdc42 (Table S1). The most interesting commonality among the 26 novel putative Cdc42 interactors is that none of them has any CRIB or CRIB-like moiety. Eleven of the sequences were represented by more than one clone from the same library, such as PARIS-1 (prostate antigen recognized and identified by SEREX 1; five clones from brain library), cAMP-dependent protein kinase regulatory subunit RI-β (five clones each from brain library), membrane-associated guanylate kinase p55 subfamily member 5 (four clones from liver library), PRKRA (protein kinase, interferon-inducible double stranded RNA-dependent activator; three clones from brain library), BRMS1-like (breast cancer metastasis-suppressor 1-like; three clones from brain library), and APC (two clones from the brain library). Three of the sequences were represented by one or more clones from more than one type of cDNA library, such as RanBP9 (four clones from fetal brain, three clones each from ovary and testis and one clone from kidney libraries), FHOD1 (three clones from bone marrow and one each from kidney and testis libraries), myosin 9A (three clones from ovary and one clone each from fetal brain and liver libraries). The interaction of Cdc42 with FHOD1 and RanBP9 was subsequently confirmed by further experiments (unpublished data).

**Figure 1:**
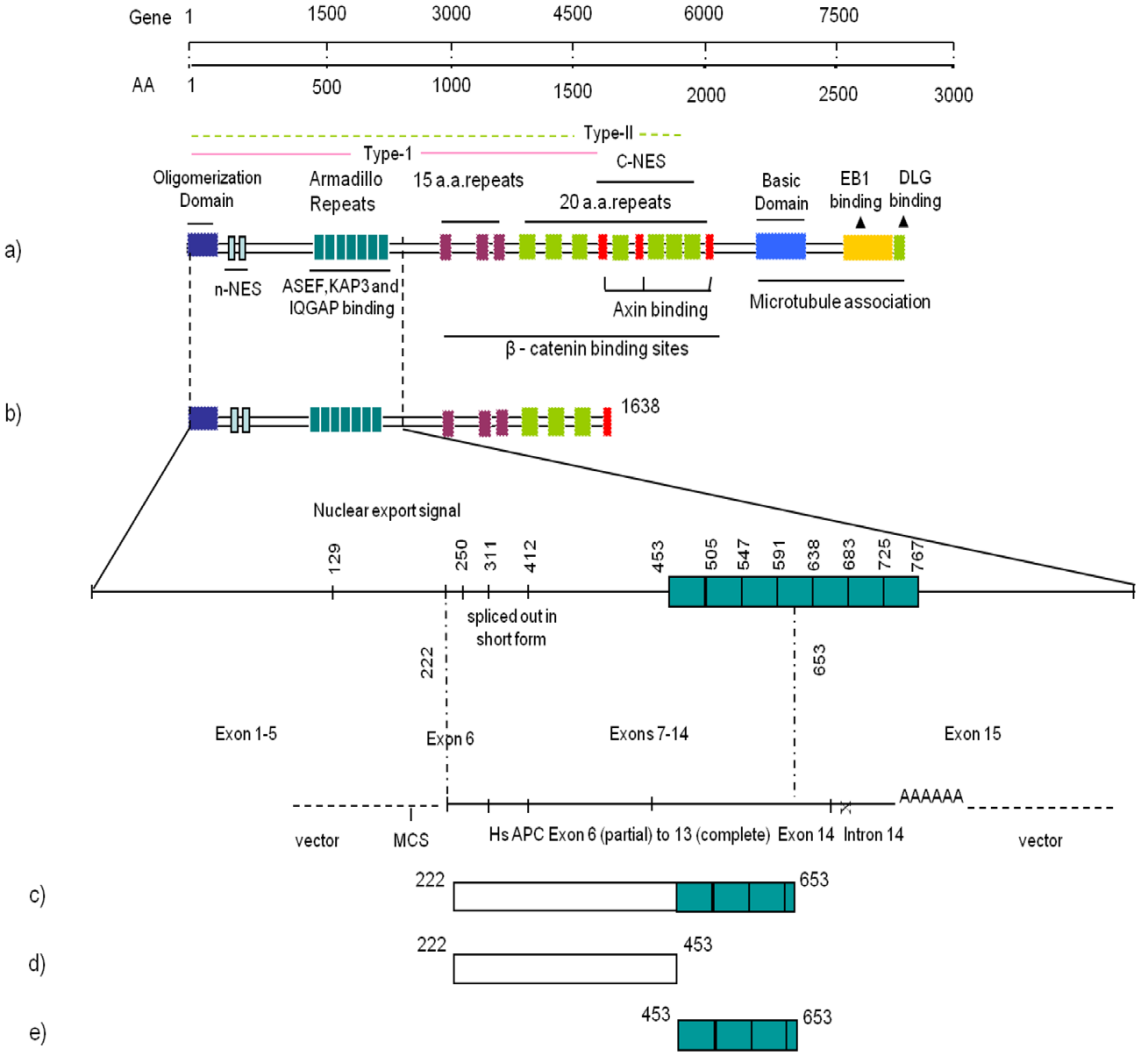
Structure of APC and its interacting constructs. Schematic domain structure of APC describing binding sites with interacting partners. (a) Full-length APC (b) APC^1–1638^ and gene structure covering the yeast two-hybrid clone (c) Yeast two-hybrid clone, APC^222–653^ (d) APC^222–453^ construct without armadillo repeats (e) APC^453–653^ construct with only armadillo repeats.

**Figure 2:**
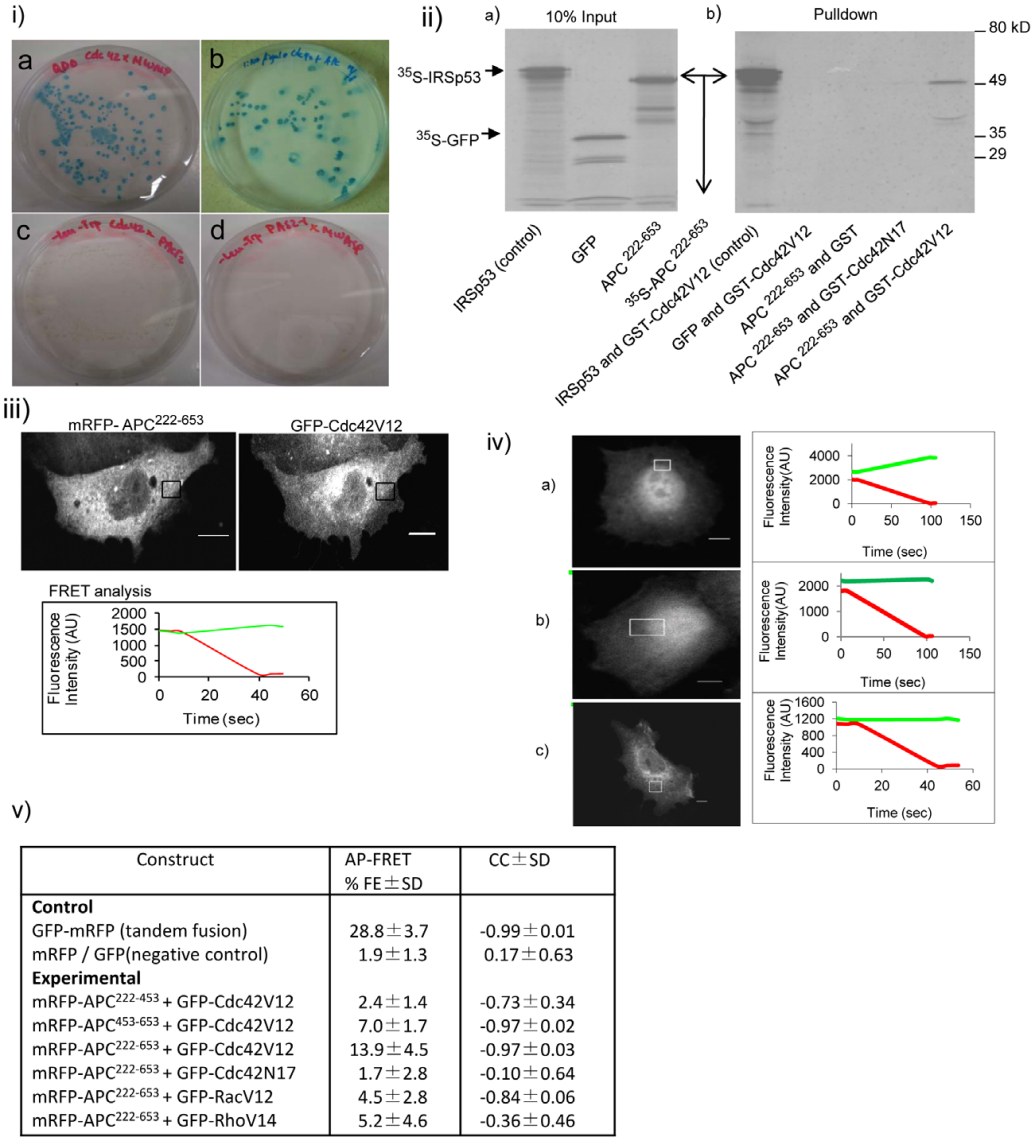
Cdc42 interacts directly with APC^222–653^ *in vitro*. (i) Cdc42-interacting APC clone from the brain cDNA library. The β-galactosidase activity of different bait and prey pairs in the Y2H screen. (a) Cdc42/N-WASP (positive control), (b) Cdc42/APC, (c) Cdc42/pACT2 (negative control) and (d) pAS2-1/N-WASP (negative control). (ii) Affinity pulldown assay. IRSp53 (positive control), GFP (negative control) and APC^222–653^ were transcribed and translated using the TNT T7 coupled reticulocyte lysate systems kit in the presence of ^35^S-methionine. The translated products were incubated with GST-tagged Cdc42V12 or Cdc42N17 or GST bound to glutathione-sepharose beads. The protein complexes were eluted by boiling and resolved by SDS-PAGE and detected by autoradiography. (a) Input for IRSp53, GFP and APC^222–653^ respectively and (b) pulldowns using different affinity columns. First two lanes are Cdc42V12 pulldowns of ^35^S-IRSp53 and ^35^S-GFP respectively and the following three lanes are GST, Cdc42N17 and Cdc42V12 pulldowns of ^35^S-APC^222–653^. (iii) APC^222–653^ interaction with Cdc42V12 as shown by AP-FRET. mRFP-APC^222–653^ and GFP-Cd42V12 were coexpressed in CHO cells and AP-FRET analysis was carried out in the ROI (box). ROI line graphs showing the FRET assay results are shown below the image. Scale bar = 5µm. (iv) AP-FRET controls. AP-FRET analysis was carried out in the ROI (box). Line graphs showing the FRET assay results are shown on the right of the respective panels. (a) cell expressing cytosolic GFP-mRFP, a tandem fusion serving as positive control, (b) cell coexpressing cytosolic GFP and mRFP serving as negative control and (c) cell coexpressing mRFP-APC^222–653^ and GFP-Cdc42N17. Scale bar = 5 µm. (v) Table shows the %FE and CC values of various APC constructs in the presence of (Cdc42V12, Cdc42N17, Rac1 or RhoA) along with positive and negative controls. Data is shown as ± SD, with three experiments and n = 7–10 cells for each experiment.

Selected clones from the brain and testis libraries encoding 10 putative novel Cdc42 interactors and one known Cdc42 binding partner were purified and used in complementation assays with Cdc42 to confirm the interactions in a clean yeast background. Among the clones was one of two identical clones encoding a 431 aa fragment of APC (APC^222–653^) that was found to interact with Cdc42 in the Y2H library screens. The exact location of the clone within the APC cDNA is shown in Fig 1. N-WASP, a known interactor of Cdc42, was included as a positive control, and bait and prey vectors lacking any inserts were used as negative controls. Matings for all clones yielded diploids that formed colonies with a consistent pattern, with the number of colonies decreasing with increasing stringency of the selection medium (QDO being the most stringent, and leucine-deficient SD medium being the least stringent). Both negative controls formed diploids that grew on SD medium lacking leucine and tryptophan but not on QDO medium (Fig. S1 (ii)). Diploids derived from the remaining clones survived on QDO medium (Fig. S1 (ii)). β-galactosidase assays were done to verify the results of the complementation assays. Colonies derived from the positive control N-WASP turned bright blue (Fig. 2(i), a) and were given color intensity scores of (+++++) while those arising from negative controls remained pale and were given a color intensity score of (+) (Fig. 2(i), c and d). APC^222–653^ gave rise to colonies that grew on QDO medium and turned blue in the β-galactosidase assay with color intensity scores of (+++++) similar to that obtained for the positive control (Fig. 2(i), b). This shows that the Cdc42-APC binding is comparable to that of Cdc42-N-WASP, and supports the view that Cdc42 interacts directly with APC^222–653^. We further investigated the interaction between Cdc42 and APC in this study.

APC is large protein comprising 2843 aa and multiple protein binding sites. The APC Y2H clone ends in intron 14 and possesses a polyA tail. It is possible that the human brain cDNA used to make the library was from an individual who had a truncation mutation in intron 14 of the APC gene, allowing it to be represented. The sequence of the APC clone encodes three full armadillo repeats (453–653 aa) and some N-terminal flanking sequences (222–453 aa). To confirm that Cdc42 did indeed interact with APC^222–653^, we carried out affinity pulldown experiments using an *in vitro* transcription/translation system. ^35^S-labelled IRSp53 (positive control), GFP (negative control) and APC^222–653^ (experimental) were incubated with purified GST, GST-Cdc42V12 or GSTCdc42N17 fusion protein. Cdc42N17 is a dominant negative mutant that remains GDP-bound and interacts with effectors weakly if at all. The data shown in Fig. 2 (ii) confirms that Cdc42V12 but not Cdc42N17 interacts with APC^222–653^ and IRSp53.

Next we used AP-FRET [18] to examine whether Cdc42 interacts with APC^222–653^ *in vivo*. Briefly, in order to carry out AP-FRET, fusion proteins of interacting pairs were made by linking the donor-acceptor pair of fluorescent proteins. In this case GFP served as the donor while mRFP was used as the acceptor. cDNA encoding GFP-Cdc42V12 and mRFP-APC^222–653^ was cotransfected into CHO cells and allowed to express for 24 h. An ROI was chosen, and pre-bleach images acquired. Bleaching was then carried out and images acquired during the bleaching process. Changes in intensity of GFP and mRFP were monitored during pre- and post-bleach and plotted in Fig. 2 (iii). FRET occurs if donor (GFP) intensity increases on acceptor (mRFP) bleaching.

The background subtracted intensities were used for FRET calculations. For AP-FRET analysis the change in GFP intensity after mRFP bleaching is expressed as %FE. In addition we determined a CC value for the rates of change in GFP and mRFP intensities on bleaching of mRFP. There should be a negative CC value when FRET occurs. Positive FRET is indicated by a FRET efficiency of >3% and CC>−0.7 (see [18] for complete explanation of the rationale behind AP-FRET). Further, CC values are only considered if %FE is above 3%. The intensity plot of AP-FRET for controls is given in Fig. 2 (iv). The average results of AP-FRET with Cdc42V12 or Cdc42N17 and mRFP-APC^222–653^ along with that of unfused GFP/mRFP pairs, and the tandem GFP-mRFP construct (that were used as controls to set the limits for negative and positive FRET respectively) are given in Fig. 2 (v). Cdc42V12 with mRFP-APC^222–653^ gave a %FE of 13.9% and a CC value of −0.97, indicating positive FRET. In contrast, when Cdc42N17 was used in place of Cdc42V12, the corresponding values were 1.7% and −0.1 respectively, suggesting no FRET. Thus three independent methods, yeast two-hybrid, affinity pulldown and FRET show that Cdc42 interacts directly with APC^222–653^. In addition, the FRET analysis shows that mRFP-APC^222–653^ interacts weakly with Rac1 but not with RhoA (Fig. 2, panel (v)). Taken together, these results suggest that Cdc42 interacts specifically with APC.

To further understand the role that the sequence in front of the armadillo repeats within APC^222–653^ plays in Cdc42 interaction, we made two shorter constructs: one without the armadillo repeats (APC^222–453^) and the other with only the armadillo repeats of APC^222–653^ (APC^453–653^), both tagged with mRFP. Fig. 2 (v) shows data from experiments carried out to evaluate AP-FRET between Cdc42V12, APC^222–453^ and APC^453–653^. With Cdc42 and APC^222–453^, the data do not satisfy the criteria for positive FRET as the %FE is below 3%. In contrast, the %FE is 7.0% with APC^453–653^, suggesting that the sequences within residues 222–453 are necessary but not sufficient for Cdc42V12 binding. Thus the full sequence of the APC^222–653^ fragment found in the Y2H screen is necessary for a strong Cdc42 interaction.

### Cdc42 interacts with APC and APC^1–1638^

**Figure 3:**
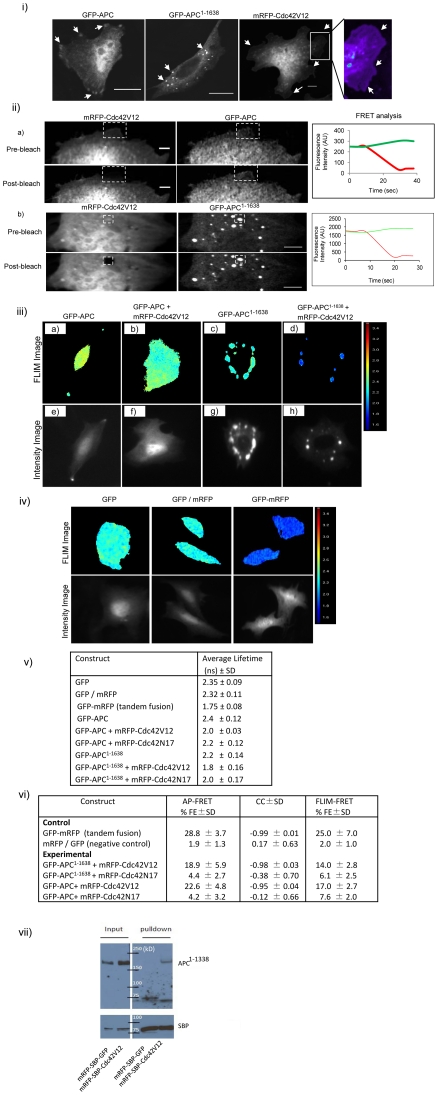
Localization and interaction of GFP-APC, GFP-APC^1–1638^ with Cdc42V12 or Cdc42N17. (i) APC localization in CHO cells. Images showing the expression and distribution of exogenous GFP-APC, GFP-APC^1–1638^ and mRFP-Cdc42V12. The enlarged image of the box in the mRFP-Cdc42V12 panel is shown on the right. This pseudo-colored image shows mRFP-Cdc42V12 localization in lamellipodia (arrow heads). Scale bar = 5µm. (ii) Cdc42V12 interacts directly with GFP-APC and GFP-APC^1–1638^ (only the leading edges of CHO cells are shown). The line graphs on the right of the images show the FRET intensity analysis. Image processing was done by applying a low pass filter in Metamorph software to subtract out-of-focus blur. (a) Cells coexpressing mRFP-Cdc42V12 and GFP-APC. AP-FRET was carried out in the ROI at the cell edges (box). Images in the top row show the pre-bleach data and those in the bottom row show post-bleach data. (b) Cell coexpressing mRFP-Cdc42V12 and GFP-APC^1–1638^. FRET analysis was carried out with vesicles as shown by the ROI. The top and bottom rows show the pre- and post-bleach images respectively. Scale bar = 5µm. (iii) FLIM-FRET images of GFP-APC and GFP-APC^1–1638^ in CHO cells. The top row shows the FLIM image of (a) GFP-APC, (b) GFP-APC and mRFP-Cdc42V12 coexpression, (c) GFP-APC^1–1638^ and (d) GFP-APC^1–1638^ and mRFP-Cdc42V12 coexpression. The respective intensity image (e–h) of each FLIM image is given in the bottom row. (iv) FLIM-FRET control images. FLIM images of CHO cells showing cytosolic GFP, cytosolic GFP/mRFP coexpression (negative control) and cytosolic GFP-mRFP tandem fusion (positive control). The intensity image of the respective FLIM image is shown in the bottom panel. (v) Table describing the average lifetime data of GFP fusion proteins in the absence and presence of mRFP-Cdc42V12 or Cdc42N17 in CHO cells. Data is shown as average lifetime ± SD from three experiments with n = 8–15 for each experiment. (vi) Table showing percentage FLIM-FRET efficiency CC values of APC and APC^1–1638^ in presence of Cdc42V12 or Cdc42N17 along with positive and negative controls. Data is shown as ± SD, with three experiments and n = 7–10 cells for each experiment. (vii) Cdc42 co-immunoprecipitation assay for endogenous APC in SW480 cells. SBP was used as an affinity tag for pulldown. cDNA encoding mRFP-SBP-GFP (negative control) or mRFP-SBP-Cdc42V12 (experimental) were separately transfected into SW480 cells and allowed to express for 24 h. After cell lysis (see Materials and Methods for details) streptavidin-sepharose beads were used to pull down SBP-tagged fusion proteins. Proteins were then resolved by SDS-PAGE and transferred to PVDF membranes. The blots were probed for APC protein using APC antibody (Ab-5 from Calbiochem) and subsequently stripped and reprobed with streptavidin-HRP antibody to assess amounts of mRFP-SBP-GFP and mRFP-SBP-Cdc42V12.

It is difficult to express full-length APC in many cell types because of its large size and its role in cell cycle arrest [24]. We found that CHO cells were a good system for this study as APC expressed well in these cells and allowed us to follow the localization of the protein. When expressed individually in CHO cells, mRFP-Cdc42V12, GFP-APC and GFP-APC^1–1638^ had distinct cellular distribution patterns (Fig. 3(i)). Full-length GFP-APC is more concentrated in the microtubule ends (Fig. S2; Dikovskaya et al. and see [25] for a review on APC localization) and throughout the cell. In contrast to full-length APC, APC^1–1638^ localized in cytoplasmic puncta as previously reported for the type II mutants [9]. Unlike full-length APC, APC^1–1638^ did not localize to the nucleus and this is likely due to the lack of a C-terminal nuclear localization sequence in the latter protein. mRFP-Cdc42V12 was localized throughout the cell with most of it in the nuclear and perinuclear area. In addition, Cdc42 expression alone induced the formation of lamellipodia [26], but this structure was not present in untransfected CHO cells. Previous studies have reported that the majority of activated Cdc42 is localized to the membrane rather than in the cytosol [27].

To determine whether Cdc42 could interact with more physiologically relevant versions of APC we carried out FRET experiments with full-length APC and a truncation mutant linked with cancer, APC^1–1638^. Cdc42 AP-FRET experiments for both APC proteins are shown in Fig. 3(ii) and Fig. 3(vi)). As suggested by the experiments showing that Cdc42V12 interacts with APC^222–653^, Cdc42V12 interacted with GFP-APC in the cytoplasm and also at the leading edges of cells. In addition, Cdc42V12 and GFP-APC^1–1638^ interacted in puncta.

The AP-FRET data was further confirmed by performing FLIM, a method where changes in the GFP fluorescence lifetime [21] of the interacting donor molecule is measured. For FLIM we used GFP and mRFP fusion proteins as donor-acceptor pairs. As expected we observed changes in GFP fluorescence lifetime when Cdc42 and APC pairs were examined (Fig. 3 (iii)). Results of control experiments with cells transfected with either GFP-mRFP fusion alone or cotransfected with unfused GFP and mRFP are given in Fig. 3 (iv). We observed that GFP-APC had an average lifetime of 2.4±0.12 ns (n = 12), but when coexpressed with mRFP-Cdc42V12 the lifetime of GFP-APC decreased to 2.0±0.03 ns (n = 14). Similarly GFP-APC^1–1638^ alone had a lifetime of 2.2±0.14 ns (n = 14), within the puncta, whereas coexpression with mRFP-Cdc42 shortened its lifetime to 1.8±0.16 ns (n = 11). The difference in lifetime observed with the expression of GFP-APC and GFP-APC^1–1638^ alone could be attributed to the refractive index of its environment [28]. The coexpression of mRFP-Cdc42N17 had a smaller effect on the GFP fluorescence lifetimes of APC and APC^1–1638^. The average GFP fluorescence lifetime of transfected cells with and without cotransfection of Cdc42 mutants are given in table (Fig. 3 (v)). The percentage FLIM-FRET efficiency (see Materials and Methods for details) was calculated for the respective interactions and is given in (Fig. 3 (vi)). Importantly, both AP-FRET and FLIM-FRET show that Cdc42 interacts directly with APC and APC^1–1638^ when coexpressed together.

### Cdc42V12 induces translocation of GFP-APC^1–1638^ to the golgi-lysozome pathway

**Figure 4:**
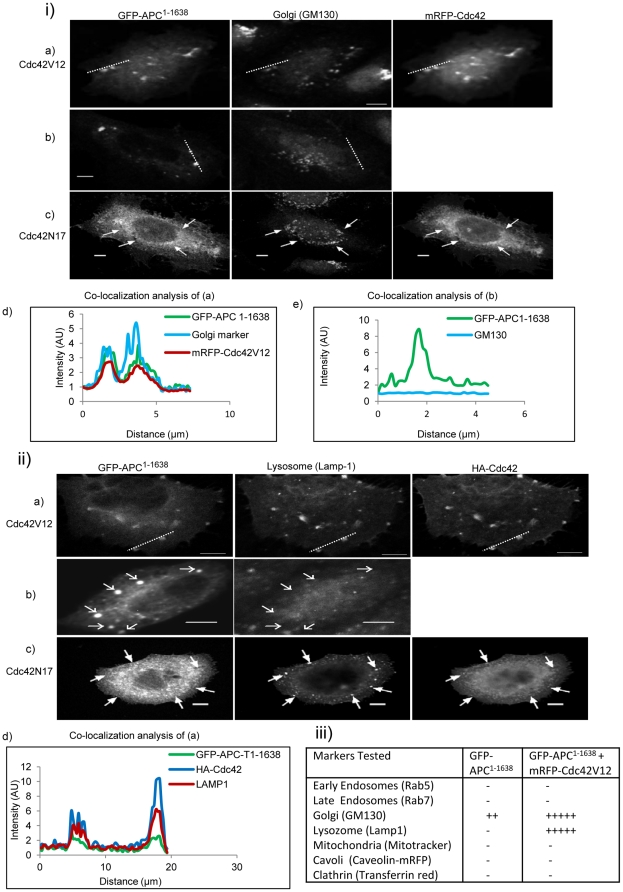
Colocalization of APC^1–1638^ in the absence and presence of Cdc42V12 with endocytic markers in CHO cells. (i) Cells were stained for golgi with GM130 primary and Alexa 405-tagged secondary antibodies. (a) Cells coexpressing GFP-APC^1–1638^ and mRFP-Cdc42V12, (b) cells expressing GFP-APC^1–1638^ (control), (c) cells coexpressing GFP-APC^1–1638^ and mRFP-Cdc42N17. The arrows mark positions for possible colocalization. Colocalization analysis was carried out along the white dotted lines as shown on the images. (d) Line intensity colocalization analysis of GFP-APC^1–1638^ and Cdc42V12 in the cell shown in (a). (e) Line intensity colocalization analysis of GFP-APC^1–1638^ and GM130 in the cell shown in (b). (ii) Cells were stained for lysozomes and Cdc42 using Lamp1 and anti-HA primary antibodies and Cy 5 and Alexa 405 secondary antibodies respectively. (a) Cells coexpressing GFP-APC^1–1638^ and HA-Cdc42V12, (b) cells expressing GFP-APC^1–1638^ (control) and (c) cells coexpressing GFP-APC^1–1638^ and HA-Cdc42N17. The arrows in (b) and (c) mark positions for possible colocalization. Colocalization analysis was carried out along the white dotted lines shown on the images. (d) Line intensity colocalization analysis of GFP-APC^1–1638^ and HA-Cdc42V12 in the cell shown in panel (a). Scale bar = 5µm. (iii) Table showing details of markers tested for GFP-APC^1–1638^ in the absence (first column) and in the presence of Cdc42V12 (second column). The plus sign shows the extent of colocalization with − being absent, ++ being the lowest and +++++ the highest.

To determine the nature of the puncta where GFP-APC^1–1638^ was localized to, we used the following markers of membrane compartments: Rab5, Rab7, GM130, Rab11, Lamp1, mitotracker, caveolin-mRFP, and transferrin red. Quantitative colocalization of GFP-APC^1–1638^ with these markers was carried out with both line intensity and ROI. GFP-APC^1–1638^ did not colocalize with any of these markers (Fig. 4 (iii)). However, in some cells GFP-APC^1–1638^ did show a partial colocalization with the golgi marker GM130. In the presence of Cdc42V12, APC^1–1638^ puncta are colocalized with GM130 (Fig. 4 (i)) and the lysozome marker Lamp1 (Fig. 4 (ii)), suggesting that Cdc42 influences GFP-APC^1–1638^ localization. The ROI colocalization analysis allows quantification of the colocalization by deriving a Pearson correlation coefficient (CC*) value. For GFP-APC^1–1638^ and GM130 marker in the presence of Cdc42V12, the CC* value was 0.66±0.14 (n = 7). Similarly the CC* value for Lamp1 and GFP-APC^1–1638^ in the presence of Cdc42V12 was 0.84±0.06 (n = 10). These results suggest that Cdc42 stimulates the GFP-APC^1–1638^ to enter a trafficking pathway that may lead to its degradation. Endogenous Cdc42 has previously been shown to localize to the golgi apparatus [29].

### Cdc42 induces translocation of GFP-APC to F-actin sites at the leading edge

**Figure 5:**
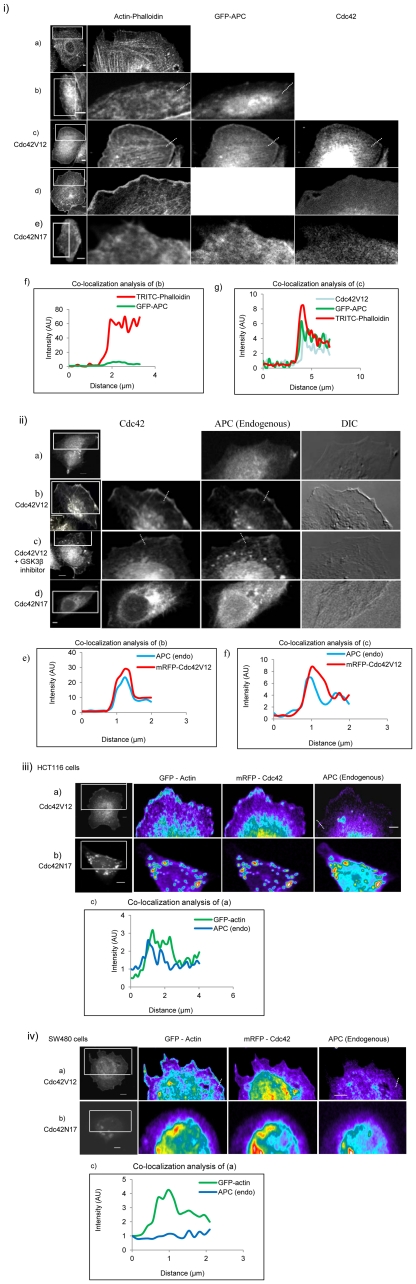
Leading edge localization of APC and Cdc42V12. (i) APC and Cdc42V12 colocalize with actin at the leading edges in CHO cells. Cells were stained with TRITC-phalloidin for actin and anti-HA for Cdc42 with Alexa 405-tagged secondary antibody. (a) untransfected CHO cell, (b) cell expressing GFP-APC, (c) cell coexpressing GFP-APC and HA-Cdc42V12, (d) cell expressing mRFP-Cdc42V12 and (e) cell expressing GFP-APC and HA-Cdc42N17. Image processing for leading edge localization was done using ImageJ software. Colocalization analysis was carried out along the white dotted lines as shown on the images. (f) Line intensity colocalization analysis of GFP-APC and TRITC-phalloidin of the cell shown in panel (b) and (g) line intensity colocalization analysis of GFP-APC, HA-Cdc42V12 and TRITC-phalloidin of the cell shown in panel (c). Scale bar = 5 µm. (ii) Endogenous APC colocalizes with Cdc42V12 at the leading edges in HCT116 cells. (a) Cell stained for endogenous APC with anti-APC primary antibody (ab-2) and Alexa 488-tagged secondary antibody, (b) cell expressing mRFP-Cdc42V12 and stained for endogenous APC, (c) mRFP-Cdc42V12 expressing cell was treated with GSK-3β inhibitor (cell-permeable PKC ζ–specific pseudosubstrate) followed by immunostaining for APC, (d) cell expressing mRFP-Cdc42N17 stained for endogenous APC. Image processing for leading edge localization was done by applying a low pass filter in Metamorph software to subtract out-of-focus blur. Colocalization analysis was carried out along the white dotted lines shown on the images. (e) Line intensity colocalization analysis of endogenous APC and mRFP-Cdc42V12 of the cell shown in panel (b) and (f) line intensity colocalization analysis of endogenous APC and mRFP-Cdc42V12 of the cell shown in panel (c). Scale bar = 5µm. (iii) Endogenous APC colocalization with GFP-actin in HCT116 cells in presence of Cdc42V12 or Cdc42N17. (a) Cell coexpressing mRFP-Cdc42V12 and GFP-actin. (b) Cell coexpressing mRFP-Cdc42N17 and GFP-actin. Image processing for leading edge localization was done by applying a low pass filter in Metamorph software to subtract out-of-focus blur. Pseudo-coloring was applied using Metamorph software. Colocalization analysis was carried out along the white dotted lines shown on the images. (c) Line intensity colocalization analysis of stained endogenous APC and GFP-actin of cell shown in panel (a). Scale bar = 5 µm. (iv) Endogenous APC colocalization with GFP-actin in SW480 cells in presence of Cdc42V12 or Cdc42N17. (a) Cell coexpressing mRFP-Cdc42V12 and GFP-actin, (b) cell coexpressing mRFP-Cdc42N17 and GFP-actin. Image processing for leading edge localization was done by applying a low pass filter in Metamorph software to subtract out-of-focus blur. Pseudo-coloring was applied using Metamorph software. Colocalization analysis was carried out along the white dotted lines shown on the images. (c) Line intensity colocalization analysis of stained endogenous APC and GFP-actin of cell shown in panel (a). Scale bar = 5µm.

In control untransfected CHO cells, most of the F-actin was in the form of stress fibers, with some also at the leading edge (Fig. 5 (i) panel a). When Cdc42V12 was expressed in CHO cells, membrane ruffles and lamellipodia were seen at the leading edge and stress fibers were reduced (Fig. 5(i) panel d). When GFP-APC was expressed on its own it had no effect on actin distribution (Fig. 5(i) panel b and f). While GFP-APC expressing cells appeared to show a decrease in stress fibers, a careful analysis of cells (n = 7) in the absence and presence of GFP-APC revealed that there was no reduction in stress fibers. When GFP-APC was coexpressed with Cdc42V12 in CHO cells, F-actin was seen at the leading edge, although this was not as pronounced as in the case of Cdc42V12 alone; membrane ruffling also appeared to be reduced (Fig. 5 (i) panel c). Interestingly, Cdc42V12 induced APC to localize to the leading edge, whereas Cdc42N17 did not (Fig. 5 (i), compare panels c and e). Using line intensity analysis we found that GFP-APC only colocalized with F-actin at the leading edge in the presence of Cdc42V12 (Fig. 5(i) panel g). Next we carried out a quantitative ROI analysis of GFP-APC with F-actin in the presence of Cdc42V12. GFP-APC and F-actin colocalized with a CC* value of 0.87±0.05 (n = 4). This suggests that APC is localized at the leading edges of cells along with actin in the presence of Cdc42.

### Effect of Cdc42 on endogenous APC proteins in colon cancer cell lines

To examine the effect of Cdc42V12 on APC localization in a more physiological context we decided to study these two proteins in two colon cancer cell lines. HCT116 cells express full-length APC while SW480 cells express a truncated form of APC (APC^1–1338^) endogenously. When we expressed mRFP-Cdc42V12, it induced the translocation of full-length APC to the leading edge in HCT116 cells as seen by a line intensity analysis (Fig. 5(ii) panels a, b and e) but mRFP-Cdc42N17 did not (Fig. 5(ii) panel d). Furthermore, a quantitative ROI analysis gave a CC* value of 0.95±0.12 (n = 8) for Cdc42V12 and endogenous APC. To rule out the possibility that relocalization of APC to the leading edge was due to GSK-3β mediated phosphorylation via the Cdc42-Par3/Par6/PKC pathway rather than direct interaction with Cdc42, we used a GSK-3β inhibitor (cell permeable PKCζ–specific pseudosubstrate) [19]. The GSK-3β inhibitor did not influence the Cdc42-induced translocation of APC to the leading edge (Fig. 5 (ii) panels c and f). The CC* value for Cdc42V12 and APC in the presence of GSK-3β inhibitor was 0.87±0.04 (n = 8).

Next we investigated the effect of Cdc42V12 on localization of endogenous APC proteins expressed in HCT116 cells and SW480 cells with actin in the presence of Cdc42V12 or Cdc42N17. In HCT116 cells, actin and APC were found to colocalize at the leading edge in presence of Cdc42V12 as shown by line intensity analysis (Fig. 5(iii) panels a and c), whereas in the presence of Cdc42N17 there was no leading edge localization seen (Fig. 5(iii) panel b). The quantitative ROI analysis gave a CC* value of 0.60±0.01 (n = 8) for APC and actin in presence of Cdc42V12. However, in SW480 cells, APC and actin did not show any colocalization at the leading edges, as evident from line intensity analyses (Fig. 5(iv) panels a and c) in presence of Cdc42V12 or Cdc42N17 (Fig. 5(iv) panel b). The quantitative ROI analysis of SW480 cells gave a CC* value of 0.33±0.10 (n = 8) for APC and actin. Only full-length endogenous APC localized to the leading edge with actin in the presence of Cdc42V12.

## Discussion

In this study we have carried out an extensive Y2H analysis of nine tissue cDNA libraries using Cdc42 as a bait. In our Y2H screens we identified most of the known Cdc42 interactors and these include IRSp53, PAK isoforms, Cdc23, ACK1 and N-WASP. We reconfirmed the Y2H results by purifying prey plasmids and introducing them into fresh yeast cells and measuring growth on QDO plates and β-galactosidase activity. Most importantly, we identified APC as a Cdc42 target. For FHOD1 and RanBP9 we have independent evidence that these proteins can bind Cdc42 (unpublished data). We believe all 10 novel partners interact specifically with Cdc42. Further analysis will be required to confirm this. Interestingly, with these 10 proteins we could not identify a common motif or resemblance to known Cdc42-binding motifs such as CRIB and HR1 and this may suggest novel Cdc42 interaction mechanisms. Our results suggest that Cdc42 affects a wider range of signaling pathways than previously thought.

APC is a large multidomain 2834 aa scaffold protein having interaction sites that take it to different cellular locations (for review see [30]). APC has been detected in the nucleus, associated with microtubules, actin-based membrane ruffles, actin-dependent cell-cell junctions, plasma membranes, mitochondria, growth cones and centrosomes. APC interaction partners that are known to help localize it include EB1 and Asef/IQGAP [2], [4], [5]. The latter two proteins associate with the APC armadillo repeats. Interestingly, in an exciting recent study, APC has been found to localize RNAs to cell protrusions [31]. Thus it may be the case that the Cdc42 interaction with APC helps this RNA-localization function of APC.

Cdc42V12 interacted with APC *in vitro*, as shown by Y2H experiments and affinity pull down, and *in vivo*, as shown by AP-FRET and FLIM. This is very strong evidence that Cdc42 can directly bind to APC. Cdc42V12 interacts directly with the armadillo repeats of APC^222–653^, thus both full-length and truncation mutants (e.g. APC^1–1638^) will be targets. Cdc42V12 was found to relocate full-length APC from microtubule ends to the leading edge along with F-actin. We showed for both exogenously and endogenously expressed APC that Cdc42V12 can affect its cellular distribution. The direct Cdc42-APC interaction, along with the Cdc42-Par3/Par6 complex, may well be necessary for cell polarity decisions. In animal models the APC truncation mutant APC^1–1638^ increases tumor susceptibility. APC^1–1638^ neither binds actin nor colocalizes with it. With the APC truncation mutant APC^1–1638^, Cdc42V12 induced a cellular redistribution to the golgi and lysozome compartments. Thus Cdc42 may lead to degradation of APC mutants and this may potentially increase the tumor susceptibility of the cells. The FRET or FLIM results also give spatial information on the Cdc42-APC interaction and this highlights the physiological significance of the analysis. Thus our conclusion is that the physiological function of Cdc42 interaction with APC serves to localize full length APC to the leading edge with F-actin and with APC^1–1638^ to the golgi/lysozome compartments. Future work with APC mutants that cannot bind Cdc42 will have to be done to further explore the physiological function of the Cdc42-APC interaction.

In preliminary experiments, we have found that Cdc42 and Asef compete with each other to bind APC (unpublished data). This could suggest that the APC-Asef interaction serves to activate Cdc42 near APC, and once activated, Cdc42 can bind APC, directing it to the leading edge with F-actin. Understanding the link between APC and the actin cytoskeleton would be a major step forward in understanding the biology of APC and its tumor suppressor activity. The well studied APC binding proteins linked to the actin machinery are Asef and IQGAP1. APC may also be associated indirectly to actin through beta-catenin. However, CHO cells lack any endogenous or detectable levels of IQGAP, Cdc42 or beta-catenin [32]–[34]. Cdc42 induced localization of APC to F-actin at the leading edge was insensitive to the GSK-3β inhibitor suggesting that the direct interaction of Cdc42 with APC is important. In addition Dikovskaya [25] have suggested that microtubule binding of APC via the basic domain is regulated by other regions of the molecules in full-length APC. Consistent with this idea our data support the proposed role of Cdc42 binding at the N-terminus of APC, linking it to F-actin and localization it to the leading edge. Cdc42 affected the localization of transfected APC in CHO cells, and endogenous APC in SW480 and HCT116 cells, suggesting that Cdc42-APC interactions have roles in both normal physiology and tumorigenesis.

RhoGTPases are linked to cancer progression and have documented roles in cell invasiveness and metastasis [35], [36]. It has been reported that in epithelial cells the disruption of polarity caused cell invasion into the surrounding environment [37]. Cdc42 has an important role in cell polarity, perhaps through its interaction with APC and actin at the leading edge [19]. Selective depolarization of basolateral membrane proteins by functional deletion of Cdc42 leads to inhibition of membrane traffic to the basolateral plasma membrane in both the endocytic and secretory pathways [38]. Further work investigating Cdc42 interaction with APC and its mutants on SW480 cells will likely reveal novel insights into the biology and cancer-causing abilities of these proteins.

## Supporting Information

Figure S1 **Y2H library screens.** (i) The pie chart shows the proportion of Cdc42-interacting clones obtained in cDNA libraries derived from nine different tissues. The cDNA libraries were screened using Cdc42Q61L/C189S as bait. (ii) The table shows growth on selection media and β-galactosidase activity of clones in Y2H complementation assays. Prey plasmid DNA encoding putative Cdc42 interactors was purified from the positive clones and co-transformed with bait plasmids into a fresh yeast background to confirm interactions. Data from human brain and testis libraries are presented. In total 14 clones were analyzed, including a known interactor, 10 novel clones and 3 controls. Numbers shown are of colonies detected with the different bait/prey pairs under different selection conditions. The plus sign shows the intensity of X-gal staining (β-galactosidase activity) with + being the lowest intensity and +++++ the highest.
(TIF)

Figure S2 **GFP-APC colocalizes with α-tubulin.** CHO cells transfected with GFP-APC were stained for α-tubulin using mouse anti α-tubulin primary antibody and anti-mouse Alexa 568-tagged secondary antibody. Scale bar = 5µm.
(TIF)

Table S1 **Yeast two-hybrid identification of putative novel Cdc42 interactors.** Sequences from 61 clones were matched with entries using BLAST and found to encode a total of 26 putative novel Cdc42 interactors not previously known to bind Cdc42 in the NCBI non-redundant nucleotide sequence database. Of these, 11 were represented by more than one clone, and 3 represented by clones from more than one type of cDNA library.
(DOC)
