# Supplementary figures and images for: Rho GTPase Cdc42 Is a Direct Interacting Partner of Adenomatous Polyposis Coli Protein and Can Alter Its Cellular Localization
Source: PLoS One. 2011 Feb 2;6(2):e16603. doi: 10.1371/journal.pone.0016603 (PMC3032772; doi:10.1371/journal.pone.0016603)

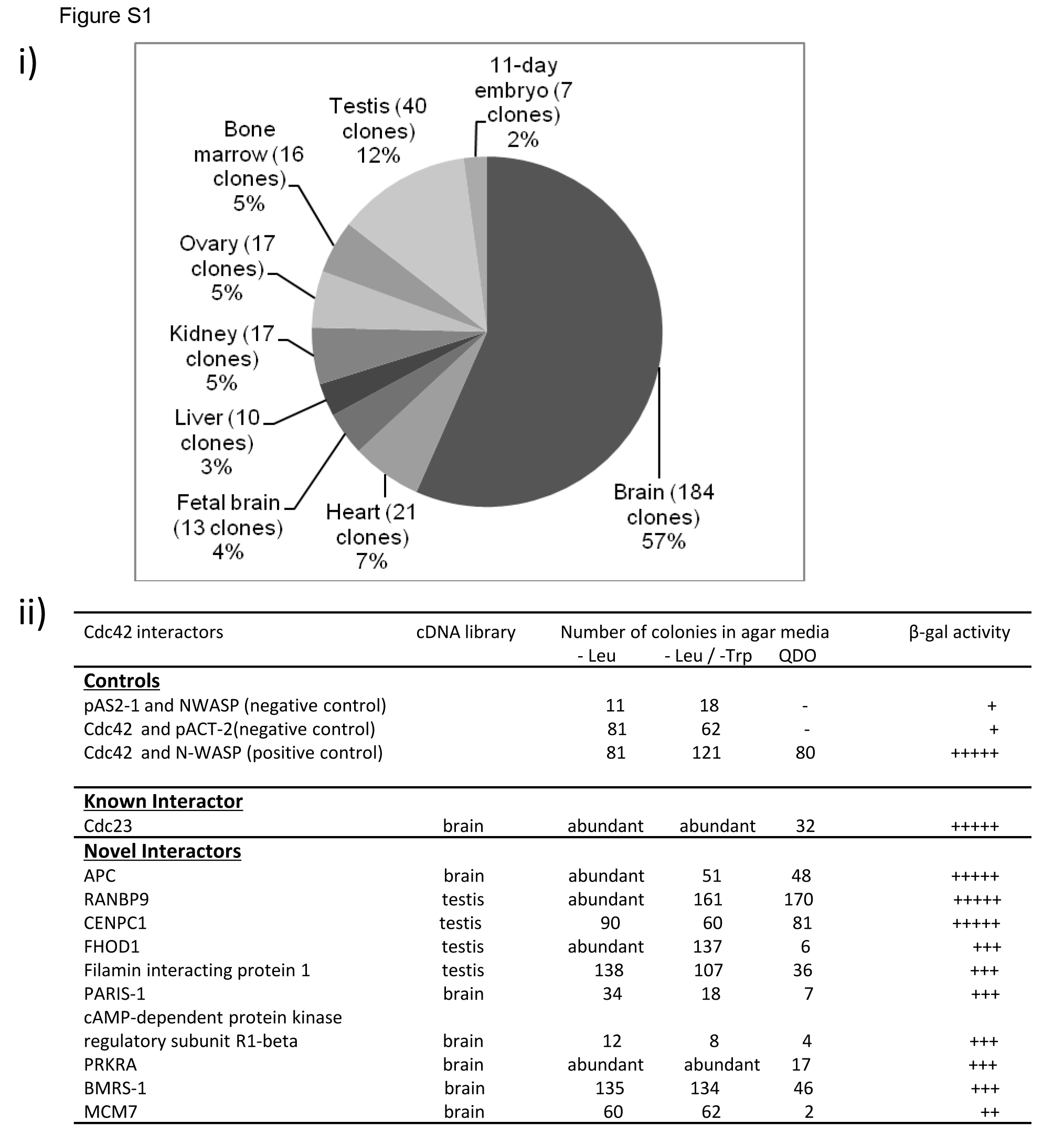

Supplement: Figure S1 — Y2H library screens. (i) The pie chart shows the proportion of Cdc42-interacting clones obtained in cDNA libraries derived from nine different tissues. The cDNA libraries were screened using Cdc42Q61L/C189S as bait. (ii) The table shows growth on selection media and β-galactosidase activity of clones in Y2H complementation assays. Prey plasmid DNA encoding putative Cdc42 interactors was purified from the positive clones and co-transformed with bait plasmids into a fresh yeast background to confirm interactions. Data from human brain and testis libraries are presented. In total 14 clones were analyzed, including a known interactor, 10 novel clones and 3 controls. Numbers shown are of colonies detected with the different bait/prey pairs under different selection conditions. The plus sign shows the intensity of X-gal staining (β-galactosidase activity) with + being the lowest intensity and +++++ the highest. (TIF) [file pone.0016603.s001.tif]

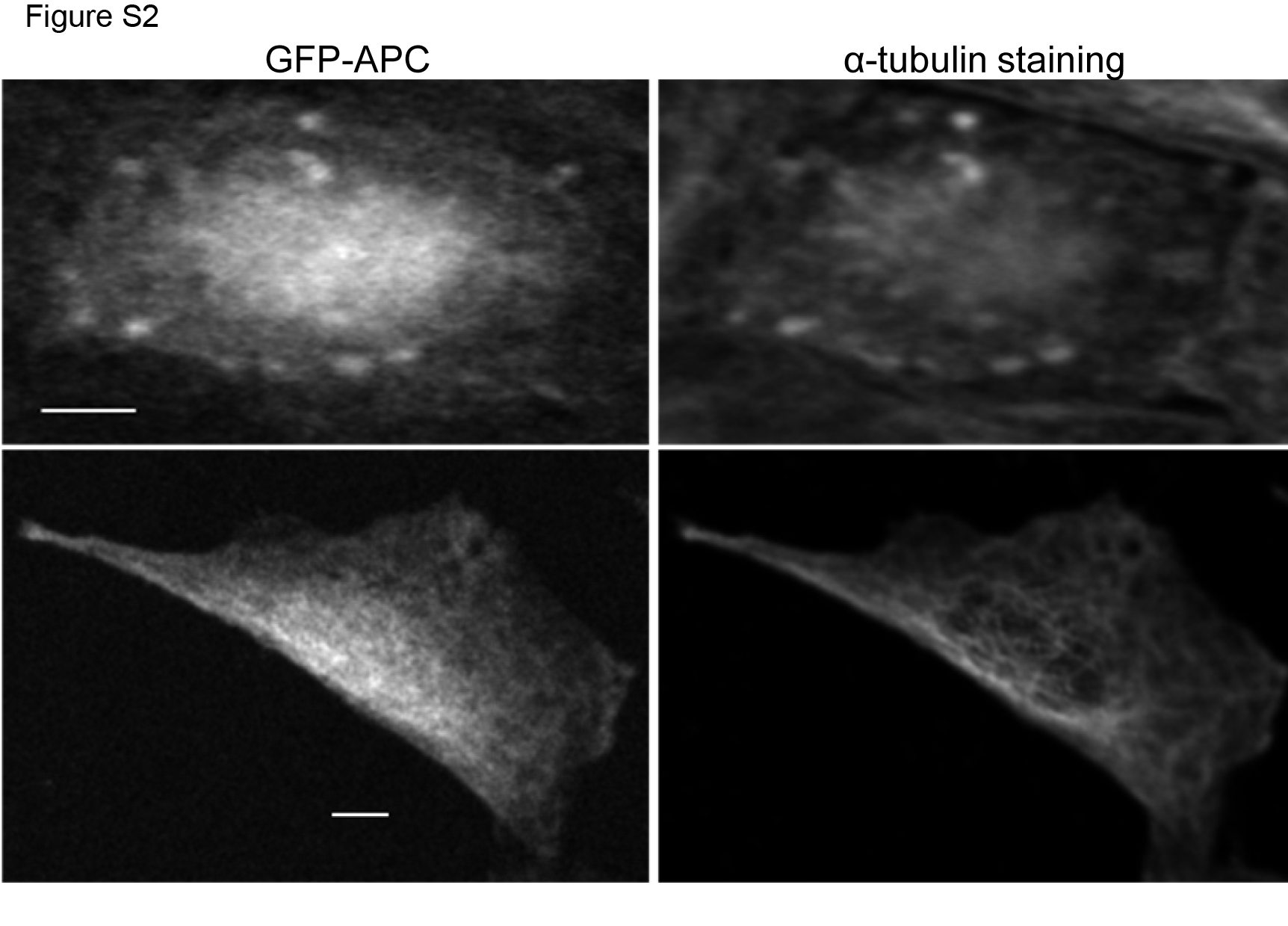

Supplement: Figure S2 — GFP-APC colocalizes with α-tubulin. CHO cells transfected with GFP-APC were stained for α-tubulin using mouse anti α-tubulin primary antibody and anti-mouse Alexa 568-tagged secondary antibody. Scale bar = 5µm. (TIF) [file pone.0016603.s002.tif]
