# Supplementary material for: Rho GTPase Cdc42 Is a Direct Interacting Partner of Adenomatous Polyposis Coli Protein and Can Alter Its Cellular Localization
Source: PLoS One. 2011 Feb 2;6(2):e16603. doi: 10.1371/journal.pone.0016603 (PMC3032772; doi:10.1371/journal.pone.0016603)
Supplement: Table S1 — Yeast two-hybrid identification of putative novel Cdc42 interactors. Sequences from 61 clones were matched with entries using BLAST and found to encode a total of 26 putative novel Cdc42 interactors not previously known to bind Cdc42 in the NCBI non-redundant nucleotide sequence database. Of these, 11 were represented by more than one clone, and 3 represented by clones from more than one type of cDNA library. (DOC) [file pone.0016603.s003.doc]

**Table S1**

|  | | | | | |
| --- | --- | --- | --- | --- | --- |
| **Putative novel Cdc42Hs interactor** | | **UniProt ID** | **Library** | **No. of clones** | **Total no. of clones** |
| RanBP9 | Q96S59 | | testis | 3 | 11 |
|  |  | | ovary | 3 |
|  |  | | fetal brain | 4 |
|  |  | | kidney | 1 |
| FHOD1 | Q9Y613 | | testis | 1 | 5 |
|  |  | | kidney | 1 |
|  |  | | bone marrow | 3 |
| Myosin 9A | Q9UNJ2 | | liver | 1 | 5 |
|  |  | | fetal brain | 1 |
|  |  | | ovary | 3 |
| cAMP-dependent protein kinase regulatory subunit RI-beta | P31321 | | brain | 5 | 5 |
|  |  | |  |  |  |
| PARIS-1 | Q9BYX2 | | brain | 5 | 5 |
| MAGUK p55 subfamily membrane 5 | Q8N3R9 | | liver | 4 | 4 |
| Breast cancer metastasis-suppressor 1-like | Q9HCU9 | | brain | 3 | 3 |
|  |  | |  |  |  |
| Protein kinase, interferon-inducible double stranded RNA dependent activator (PRKRA) | O75569 | | brain | 3 | 3 |
| 26S protease regulatory subunit 6B | P43686 | | brain | 2 | 2 |
| Adenomatous polyposis coli | P25054 | | brain | 2 | 2 |
| ARHGEF3 | Q9NR81 | | brain | 1 | 1 |
| Centromere protein C1 | Q03188 | | testis | 1 | 1 |
| Component of oligomeric golgi complex 1 | Q8WTW3 | | testis | 1 | 1 |
| Connective tissue growth factor | Q6FHL8 | | testis | 1 | 1 |
| Cytokine-like nuclear factor n-pac | Q49A26 | | heart | 1 | 1 |
| Filamin A interacting protein 1 | Q7Z7B0 | | testis | 1 | 1 |
| Glucocorticoid receptor DNA binding factor 1 (GRLF1) | Q9NRY4 | | brain | 1 | 1 |
| HtrA serine peptidase 1 (HTRA1) | Q92743 | | kidney | 1 | 1 |
| Minichromosome maintenance deficient 7 | P33993 | | brain | 1 | 1 |
| mFLJ00128 protein | Q6KAS1 | | 11 day embryo | 1 | 1 |
| *Mus musculus* SH3 domain containing ring finger 1 | O70254 | | 11day embryo | 1 | 1 |
| Polycomb group ring finger 6 | Q9BYE7 | | testis | 1 | 1 |
| Puratrophin-1 | Q58EX7 | | testis | 1 | 1 |
| TBC1 domain family, member 4 | O60343 | | brain | 1 | 1 |
| Upstream transcription factor 2, c-fos interacting | Q15853 | | kidney | 1 | 1 |
|  |  | |  |  |  |
| Zinc finger, MYND domain containing 11 | Q15326 | | brain | 1 | 1 |
| **Total** |  | |  |  | **61** |
